# Establishing a Risk Prediction Model for Nasopharyngeal Carcinoma Based on Anti-BNLF2b Serological Biomarkers: A Retrospective Study

**DOI:** 10.7150/ijms.110758

**Published:** 2025-04-13

**Authors:** Hengrong Shao, Mengting Chen, Yufei Xiao, Li Xu, Huaquan Cao, Bo Hong, Yun Qian

**Affiliations:** 1 Department of Clinical Laboratory, Stomatology Hospital, School of Stomatology, Zhejiang University School of Medicine, Zhejiang Provincial Clinical Research Center for Oral Diseases, Key Laboratory of Oral Biomedical Research of Zhejiang Province, Cancer Center of Zhejiang University, Hangzhou 310000, China.; 2 Department of Clinical Laboratory, The Second Affiliated Hospital, Zhejiang University School of Medicine, Hangzhou 310009, China.; 3 Department of Pathology, The Second Affiliated Hospital, Zhejiang University School of Medicine, Hangzhou 310009, China.

**Keywords:** Nasopharyngeal carcinoma, Epstein-Barr virus, Serum biomarker, BNLF2b

## Abstract

**Purpose:** This study aims to establish a suitable risk prediction model of NPC in regions with relatively low-incidence in southern China.

**Methods:** We retrospectively analysed the data of 198 patients with NPC and 398 healthy individuals admitted to The Second Affiliated Hospital, Zhejiang University School of Medicine, from February 2023 to October 2024. The levels of different serum biomarkers (P85-Ab, VCA-IgA, VCA-IgM, VCA-IgG, Rta-IgG and EA-IgA) were compared between patients with NPC and healthy individuals. Binary logistic regression was used to construct a risk prediction model for NPC, and ROC curves were plotted to evaluate the performance of the model.

**Results:** Compared with healthy individuals, patients with NPC exhibited significantly elevated levels of EA-IgA (P < 0.001), Rta-IgG (P < 0.001), P85-Ab (P < 0.001) and VCA-IgA (χ^2^ = 262.25; P < 0.001). Binary logistic regression showed that P85-Ab (HR = 572.225; P < 0.001), VCA-IgA (HR = 31.877; P < 0.001) and Rta-IgG (HR = 10.670; P = 0.004) were independent risk factors for NPC. The AUC of P85-Ab combined with Rta-IgG and VCA-IgA for predicting the risk of NPC was 0.977 (95% CI: 0.959-0.988), which was greater than the AUC values of Rta-IgG and VCA-IgA (P < 0.01 for all). The combination of P85-Ab with Rta-IgG and VCA-IgA had a sensitivity of 91.36% and a specificity of 99.25%.

**Conclusion:** P85-Ab combined with VCA-IgA and Rta-IgG is an optimal serological biomarker for the diagnosis of NPC in low-incidence regions in southern China.

## Introduction

Nasopharyngeal carcinoma (NPC) is a malignant epithelial tumour originating from the nasopharynx 1. The geographical distribution of NPC cases is extremely uneven worldwide, with a higher prevalence of NPC in southern China, southeast Asia and north Africa 2-4. China has the highest incidence (42.4%) and mortality (38.7%) of NPC in the world 5. Therefore, NPC remains a serious health problem in China.

In 1976, Epstein-Barr virus (EBV) infection was first reported to be associated with NPC when abnormally elevated antibody titres against specific viral antigens were detected in patients with NPC 6. EBV is a common pathogen that infects 95% of the global population; however, only a small proportion of individuals with EBV infection develops NPC 7, 8. Studies have shown that patients with early stages of NPC (stages I and II) can survive long term; however, a majority of patients (an estimated 80%) have advanced disease at diagnosis because early symptoms are nonspecific 2. Despite appropriate treatment, the 5-year survival rate of patients with advanced NPC remains 70%-80% 2, 9, 10. Therefore, identifying EBV-related biomarkers with high specificity for NPC is necessary to establish accurate, reliable and clinically applicable large-scale screening methods to improve the early diagnosis of NPC.

The life cycle of EBV is divided into two phases: latency and lytic replication. EBV nuclear antigen 1 (EBNA1) is a protein expressed during latency 4, 11, whereas viral capsid antigen (VCA), early antigen (EA) and BRLF1 transcription activator protein (Rta) are proteins expressed during lytic replication 4, 12-14. Patients with NPC have higher levels of anti-EBV antibodies, which are beneficial for early diagnosis of NPC 7, 15, 16. However, the sensitivity and specificity of detecting these antibodies vary based on geographic locations, serum markers, detection methods and NPC stages. For instance, the sensitivity and specificity of detecting Rta-IgG via enzyme-linked immunosorbent assay (ELISA) are reported to be 83.6% and 82.4%, respectively, in Shanghai 12 and 65.6% and 95.2%, respectively, in Fuzhou 17. In Zhongshan, the sensitivity and specificity of chemiluminescent immunoassay (CLIA) for detecting VCA-IgA are reported to be 91.5% and 94.4%, respectively, whereas those of ELISA are reported to be 88.6% and 92.6%, respectively 18. The sensitivity of detecting VCA-IgA via CLIA is reported to be 83.1% in early NPC (stages I and II) and 93.6% in advanced NPC (stages III and IV) 18. The sensitivity and specificity of VCA-IgG detection are reported to be 95% and 55% 19, those of EA-IgA detection are reported to be 68% and 97% 19 and those of EBNA1-IgA are reported to be 92% and 80% 20, respectively. Considering mutations in EBV genes and the heterogeneity of NPC 21-23, the simultaneous detection of multiple anti-EBV antibodies may help improve the accuracy of diagnosing NPC 24, 25. For instance, the sensitivity and specificity of the combination of EBNA1-IgA and EA-IgA for detecting NPC are reported to be 98% and 82%, respectively, in Taiwan 26. Seropositivity for any two of EBNA1-IgG, EBNA1-IgA and Zta-IgG has been shown to have a sensitivity of 92% and a specificity of 93% for detecting NPC in the Pearl River Estuary in Southern China 27. In addition, the combined detection of VCA-IgA and EBNA1-IgA has been shown to have a sensitivity of 93% and specificity of 92% for detecting NPC in Sihui and Zhongshan 28. Recently, researchers identified a highly specific serum marker for NPC, P85-Ab, which is an antibody against a 74-amino acid BNLF2b peptide fragment 29. In a large-scale screening in Zhongshan, the sensitivity and specificity of P85-Ab for detecting NPC were found to be 97.5% and 98.3%, respectively, whereas those of the combination of P85-Ab, VCA-IgA and EBNA1-IgA were found to be 70.2% and 99.8%, respectively 29. All of the abovementioned studies were conducted in regions of southern China that have a high prevalence of NPC. However, studies investigating the sensitivity and specificity of anti-EBV antibodies in regions of southern China with a relatively low incidence of NPC, such as Zhejiang, are lacking. Therefore, identifying the optimal combination of anti-EBV antibodies for detecting NPC in these regions is necessary.

In this retrospective case-control study, we compared the diagnostic efficacy of six anti-EBV antibodies for NPC in Zhejiang, a province in southern China with a relatively low incidence of NPC. The diagnostic efficacy of the novel serum marker P85-Ab and two antibodies of VCA (IgG and IgM) was evaluated via CLIA, whereas that of three other traditional serum markers was evaluated using commercially available ELISA kits. These serum markers were used to distinguish patients with histopathologically diagnosed NPC from healthy individuals and patients with other types of cancers. In addition, we established a model for predicting the risk of NPC using logistic regression.

## Methods

### Participants

In this retrospective, observational study, serum specimens were collected from 198 patients with NPC admitted to The Second Affiliated Hospital, Zhejiang University School of Medicine (Hangzhou, China), between February 2023 and October 2024. The inclusion criteria comprised patients newly diagnosed with NPC, staged according to the 8^th^ edition of the tumour-node-metastasis (TNM) classification system by the American Joint Committee on Cancer (AJCC), with stage distribution as follows: stage I (1 case), stage II (5 cases), stage III (37 cases), and stage IV (28 cases), and who had provided serum specimens prior to the initiation of any treatment. The exclusion criteria included patients who had undergone chemoradiotherapy or had other tumours at the time of sample collection. Data on age, sex, date of initial diagnosis, date of initial treatment and TNM stage were collected from the medical records of the enrolled patients. In addition, we randomly recruited 398 healthy individuals who had visited the hospital for a routine physical examination (control group). The specimens remaining after clinical tests were used after obtaining oral informed consent from the participants. This study was approved by the Ethics Committee of The Second Affiliated Hospital, Zhejiang University School of Medicine (approval no.: 2024‑0455; Hangzhou, China) and the Stomatology Hospital, Zhejiang University School of Medicine (approval no.: 2023‑047; Hangzhou, China). The Ethics Committee waived the requirement for written informed consent because of the anonymous nature of the retrospectively collected clinical data.

### Serological testing

All serum samples were stored at -40°C until further use. P85-Ab, EA-IgA, Rta-IgG, VCA-IgG, VCA-IgM and VCA-IgA were detected in all eligible participants.

EA-IgA (Tarcine BioMed Inc.), Rta-IgG (Tarcine BioMed Inc.), and VCA-IgA (EUROIMMUN) were detected via indirect ELISA using 96-well streptavidin microplates, and VCA-IgG (DiaSorin) and VCA-IgM (DiaSorin) were detected using CLIA at the Immunology Laboratory of The Second Affiliated Hospital, Zhejiang University School of Medicine. P85-Ab (Wantai BioPharm) was detected using a CLIA kit according to the manufacturer's instructions at the Clinical Laboratory of Stomatology Hospital, Zhejiang University School of Medicine (Hangzhou, China).

### Statistical analysis

All statistical analyses were performed using the SPSS (version 21.0, IBM Corp.) and GraphPad Prism (version 8.0, GraphPad Corp.) software. MedCalc (version 18.2.1, MedCalc Corp.) was used to plot ROC curves, which were used to determine the optimal cutoff values for serum biomarker levels. Continuous variables were compared using the Mann-Whitney U test, whereas categorical variables were compared using the χ^2^ or Fisher's exact probability test. A binary logistic regression model was used for multivariate analysis of variables identified as significant predictors of NPC in univariate analysis. Statistical significance was defined as a two-sided P-value of <0.05.

## Results

### Baseline characteristics of participants

**Figure 1:**
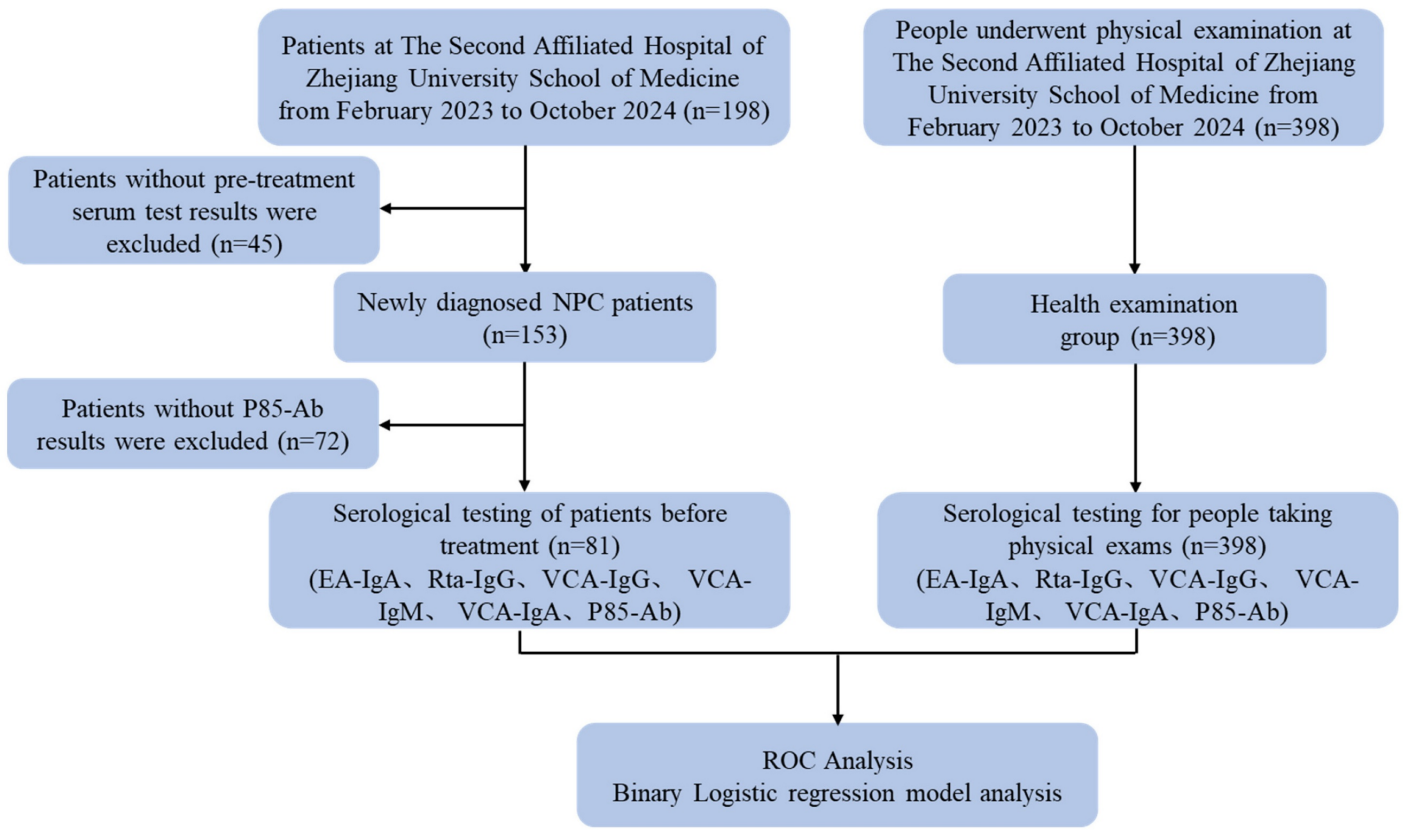
Flow chart of the criteria used to select the participants for inclusion in the present study.

A total of 198 patients with NPC were admitted to The Second Affiliated Hospital, Zhejiang University School of Medicine, between February 2023 and October 2024. A total of 81 patients with newly diagnosed NPC who met the inclusion criteria and 398 healthy individuals were eventually included in this study. A flowchart demonstrating the participant selection protocol is presented in Figure 1.

The median age of patients with NPC at diagnosis was 57 (range, 27-81) years, whereas that of healthy individuals was 47 (range, 27-88) years. A total of 364 (76%) participants were men, 309 (77.6%) in the control group and 55 (67.9%) in the NPC group. The median value of serum EA-IgA, Rta-IgG and P85-Ab were 0.61 (range, 0.03-5.11), 0.95 (range, 0.05-4.12) and 43.32 (range, 0.01-639.48), respectively, in the NPC group and 0.12 (range, 0.01-2.60), 0.19 (range, 0.03-3.22) and 0.01 (range, 0.00-13.01), respectively, in the control group. Compared with the control group, the NPC group had higher levels of EA-IgA (P < 0.001), Rta-IgG (P < 0.001), P85-Ab (P < 0.001) and VCA-IgA (χ^2^ = 262.25; P < 0.001) (Table 1). However, no significant differences were observed in the serum levels of VCA-IgG (χ^2^ = 0.669; P = 0.413) or VCA-IgM (χ^2^ = 0.953; P = 0.329) between the NPC and control groups (Table 1). Moreover, no significant differences were observed in the serum levels of any of the six markers between patients with early (stage I or II) and advanced (stage III or IV) NPC (Table 1).

### Serological biomarker levels in patients with newly diagnosed NPC, patients with other cancers and healthy individuals

**Figure 2:**
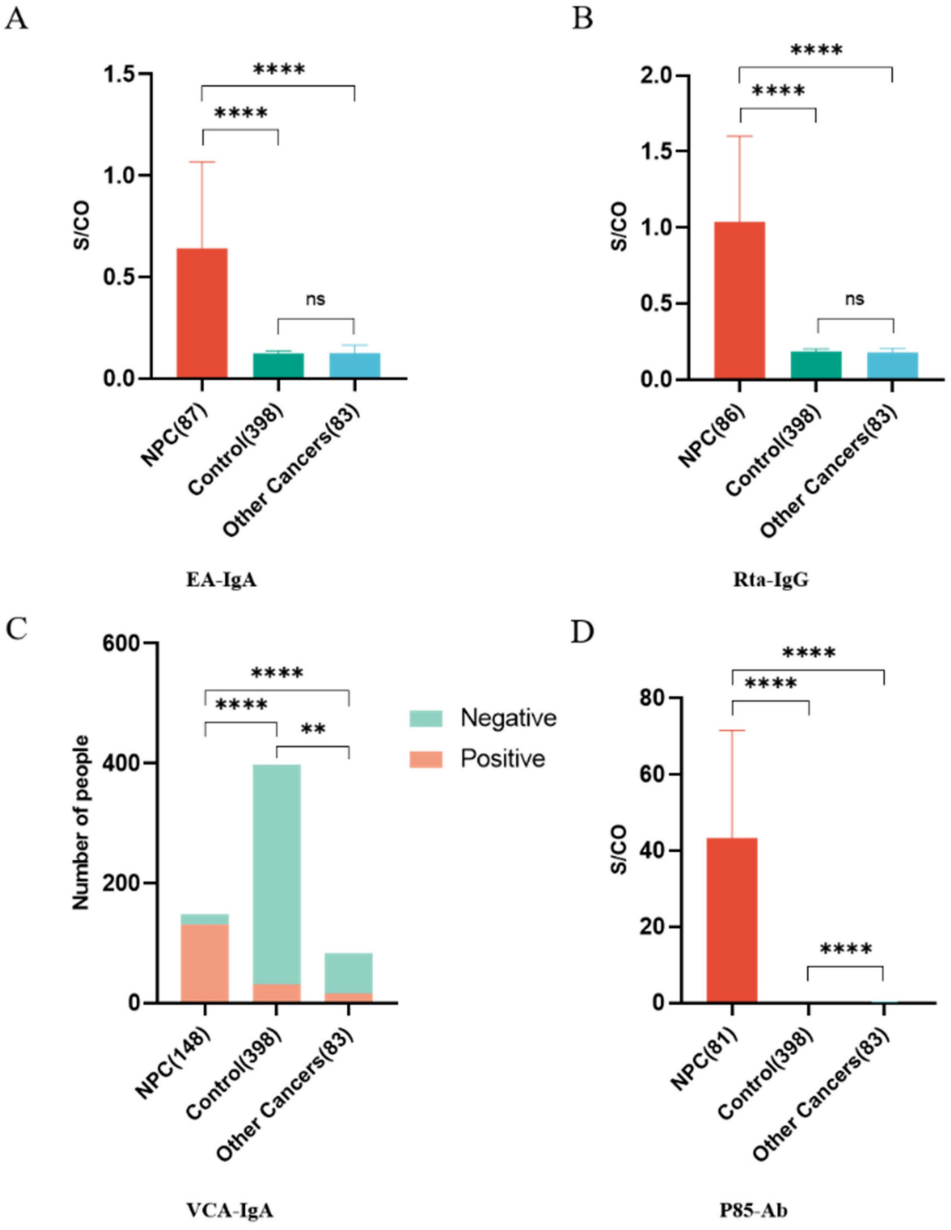
** Comparison of the levels of four serum markers (EA-IgA, Rta-IgG, VCA-IgA and P85-Ab) in NPC, control and other cancers groups**. **A** The serum levels of EA-IgA in NPC, control and other cancers groups; **B** The serum levels of Rta-IgG in NPC, control and other cancers groups; **C** The serum levels of VCA-IgA in NPC, control and other cancers groups; **D** The serum levels of P85-Ab in NPC, control and other cancers groups.

We included 83 patients with other types of cancers (such as oesophageal malignant tumours, maxillary sinus malignant tumours, sphenoid sinus malignant tumours, gastric carcinoma and lymphoma) for further comparison. The median value for serum EA-IgA, Rta-IgG and P85-Ab in these patients were 0.124 (range, 0.011-4.289), 0.179 (range, 0.036-2.685) and 0.180 (range, 0.010-0.280), respectively. The serum levels of EA-IgA, Rta-IgG, VCA-IgA and P85-Ab were significantly lower in these patients than in those with newly diagnosed NPC (P < 0.0001 for all; Figure 2). However, no significant differences were observed in the serum levels of EA-IgA (P =0.48; Figure 2A) or Rta-IgG (P =0.46; Figure 2B) between the control group and the other cancers group.

### Combined evaluation of P85-Ab, VCA-IgA, Rta-IgG and EA-IgA levels for predicting the risk of NPC

**Figure 3:**
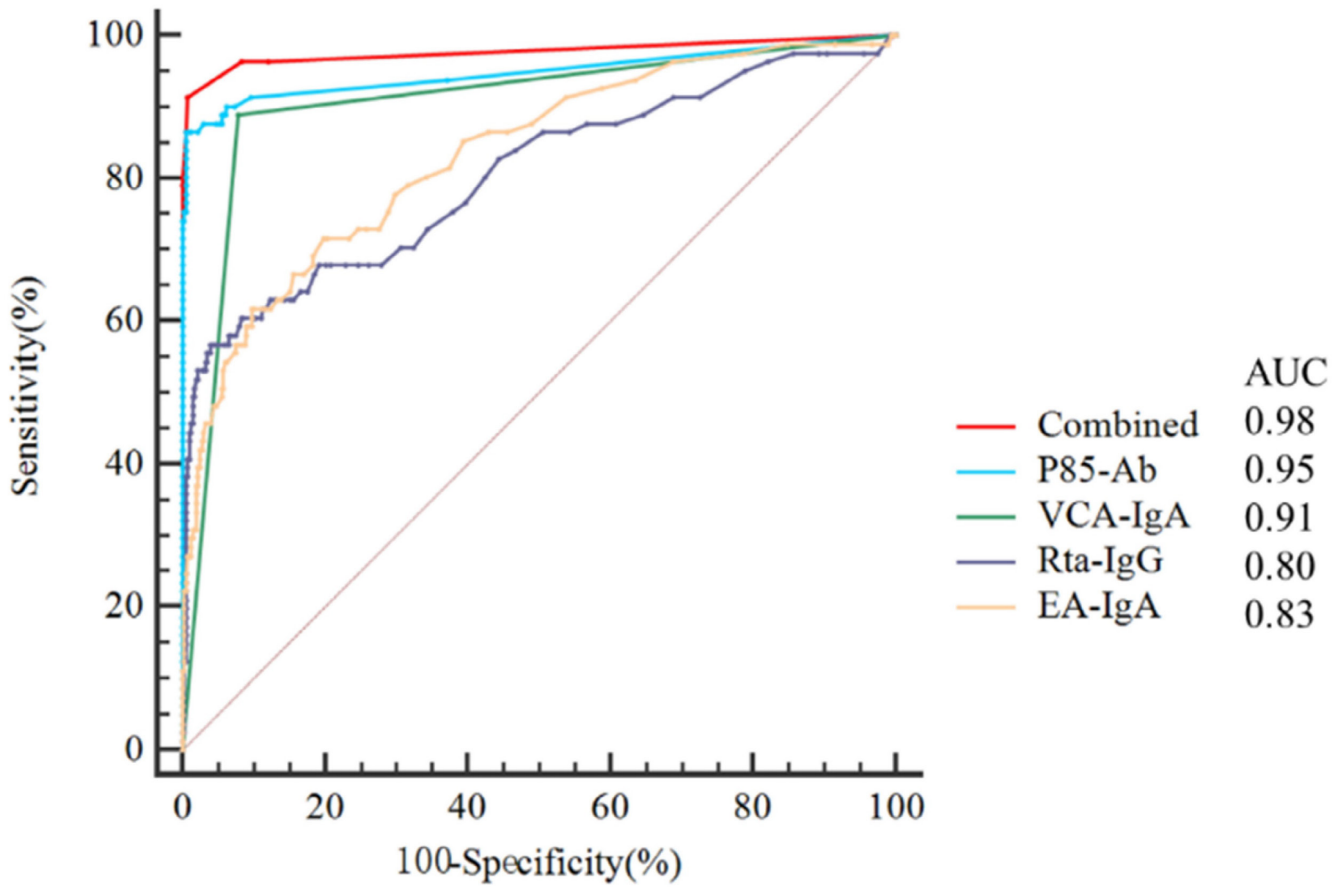
** Receiver operating characteristic curves (ROCs) for P85-Ab, VCA-IgA, Rta-IgG, EA-IgA and combination of P85-Ab, VCA-IgA and Rta-IgG.** AUC denotes area under the curve.

ROC curves were plotted to evaluate the predictive value of P85-Ab, VCA-IgA, Rta-IgG and EA-IgA levels and the combination of P85-Ab, VCA-IgA and Rta-IgG levels in the diagnosis of NPC (Figure 3). The AUC values of P85-Ab, VCA-IgA, EA-IgA and Rta-IgG for predicting the risk of NPC were 0.949 (95% CI, 0.925-0.967), 0.905 (95% CI, 0.876-0.930), 0.832 (95% CI, 0.796-0.865) and 0.804 (95% CI, 0.766-0.839), respectively (Figure 3). However, the AUC value of the combination of P85-Ab, VCA-IgA and Rta-IgG was 0.977 (95% CI, 0.959-0.988), which was larger than the individual AUC values of VCA-IgA and Rta-IgG (P < 0.0001 for all; Table 2). Although the AUC value of P85-Ab combined with VCA-IgA and Rta-IgG was greater than that of P85-Ab alone, it was not statistically significant (P = 0.088; Table 2).

### Analysis of relevant variables using binary logistic regression

**Figure 4:**
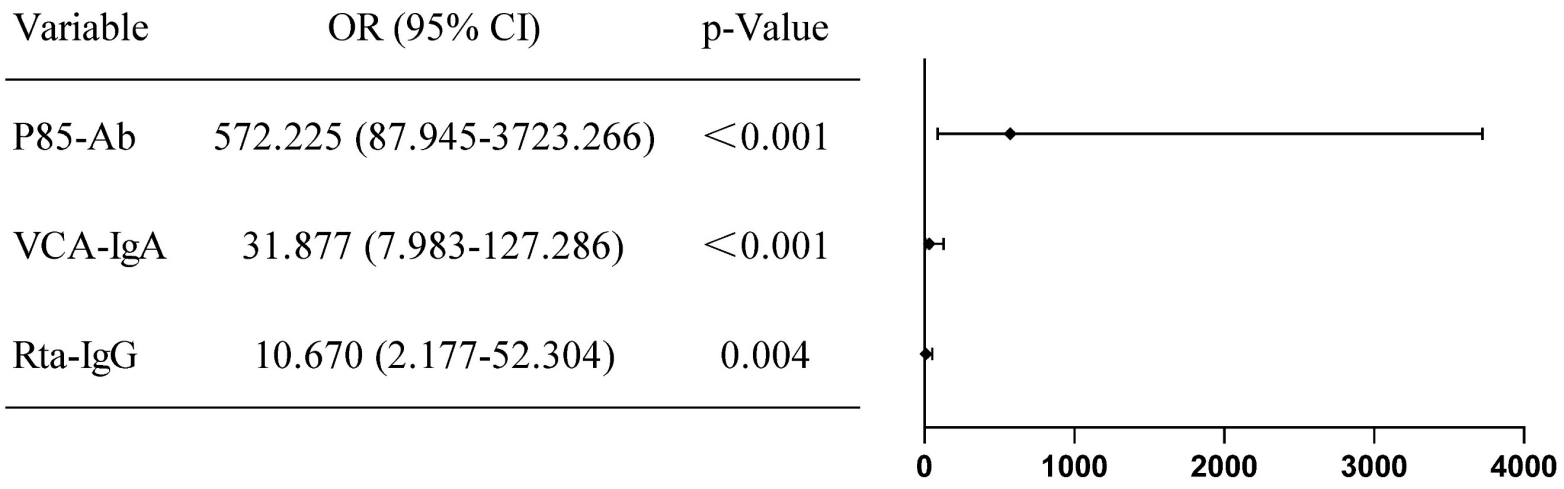
** Binary logistic risk regression model analysis was performed to analyze the risk factors associated with NPC incidence.** The odds ratios (OR) and 95% confidence intervals (CI) of risk factors associated with NPC are provided.

Binary logistic regression analysis revealed P85-Ab (HR = 572.225; P < 0.001), VCA-IgA (HR = 31.877; P < 0.001) and Rta-IgG (HR = 10.670; P = 0.004) as independent risk factors for NPC (Figure 4). A risk prediction model was developed based on these markers with the following formula: -5.214 + 6.35 × P85-Ab + 3.462 × VCA-IgA + 2.367 × Rta-IgG. The cutoff risk score was estimated to be 0.148. The sensitivity and specificity of P85-Ab, VCA-IgA, Rta-IgG and EA-IgA for predicting the risk of NPC were 86.42% and 99.50%, 88.89% and 92.21%, 56.79% and 95.98% and 61.73% and 90.20%, respectively. The combination of P85-Ab with VCA-IgA and Rta-IgG markedly increased the sensitivity to 91.36%, with a specificity of 99.25% (Table 2).

## Discussion

Although China has the highest incidence and mortality rates of NPC in the world, NPC accounts for only 0.6% of all cancer cases and 0.8% of all cancer-related deaths worldwide 5. Therefore, for large-scale NPC screening, more specific biomarkers are required to reduce the rate of misdiagnosis and the frequency of unnecessary follow-ups 13, which are time-consuming and expensive and may cause anxiety in individuals. Furthermore, serological diagnostic methods for NPC have been widely investigated in recent years and studies have shown that simultaneous detection of multiple serological markers may slightly increase diagnostic efficacy when compared with the detection of a single serological marker 13, 25. However, most studies investigating these serological markers have been conducted in southern China, where NPC is prevalent 13, 29-31. The findings of these studies may not be applicable to regions where the incidence of NPC is relatively low. Consequently, we designed this study to establish a model for predicting the risk of NPC in individuals from Zhejiang, a province in southern China with a relatively low incidence of NPC.

In this study, ELISA was used to detect three traditional EBV-related biomarkers (EA-IgA, Rta-IgG and VCA-IgA) and CLIA was used to detect a novel EBV-related biomarker (P85-Ab) and two antibodies of VCA (IgM and IgG) to establish a risk prediction model for NPC. The results showed that the sensitivity and specificity of VCA-IgA for predicting the risk of NPC were 88.89% and 92.21%, respectively; those of Rta-IgG were 56.79% and 95.98%, respectively, and those of EA-IgA were 61.73% and 90.20%, respectively. In a meta-analysis focusing on serum diagnostic biomarkers for NPC, the sensitivities of VCA-IgA, Rta-IgG, and EA-IgA were reported as 0.85, 0.70, and 0.55, respectively 32. In this study, only the sensitivity of Rta-IgG was lower than that reported in the meta-analysis, a discrepancy that may be attributed to differences in detection methods. Notably, the sensitivities of VCA-IgA and EA-IgA in this study were higher than those reported in the meta-analysis, further confirming the reliability and potential advantages of these biomarkers in the diagnosis of NPC. Additionally, since no significant differences were observed in the serum levels of VCA-IgM or VCA-IgG between the NPC and control groups, these markers were excluded from subsequent logistic regression analysis.

P85-Ab is a recently identified diagnostic biomarker for NPC. Although clinical research on P85-Ab is still limited, this study confirms its diagnostic efficacy in NPC patients, demonstrating a sensitivity of 86.42% and a specificity of 99.50%. This high specificity is beneficial for detecting NPC in low-incidence regions and is consistent with that reported in previous studies (99.6%; 95% CI, 97.8%-99.9%) 29. However, the sensitivity is lower than that reported in previous studies (94.4%; 95% CI, 86.4%-97.8%) 29. This difference may be attributed to either the small number of patients with NPC included in this study or the heterogeneity of NPC. Traditionally, the detection of serum biomarkers for NPC relies on ELISA 32. In this study, we utilized CLIA to detect the novel biomarker P85-Ab, demonstrating significantly superior diagnostic performance compared to conventional methods.

EBV is associated not only with NPC but also with Hodgkin's lymphoma, T-cell lymphoma, NK/T-cell lymphoma and some gastric cancers 33. Therefore, we included patients with lymphoma and gastric cancer who did not have NPC to validate the specificity of the four EBV-related markers. As anticipated, the serum levels of EA-IgA, Rta-IgG, VCA-IgA and P85-Ab were substantially lower in these patients than in those with NPC, which validated the specificity of these four serum markers for NPC.

Furthermore, logistic regression analysis showed that P85-Ab combined with VCA-IgA and Rta-IgG represented the optimal model for discriminating between patients with NPC and healthy individuals, with an AUC value of 0.98 (95% CI, 0.96-0.99). EA-IgA was not included in the model, which is consistent with previously reported findings that EA-IgA is suitable for the diagnosis of NPC but not for the screening of NPC 32. The combination of P85-Ab, VCA-IgA and Rta-IgG had a comparable specificity to that of P85-Ab (99.3% versus 99.5%); however, the sensitivity of the combination (91.36%) was higher than that of P85-Ab (86.42%). Consistent with the findings of existing studies, we found that the combined use of serum markers not only improved sensitivity but also maintained specificity.

EBV DNA testing has been found to be effective for NPC screening 34-36. However, real-time quantitative PCR is expensive, time-consuming and technically demanding; therefore, it was not used in this study. A unified or standardized method for EBV DNA testing is currently unavailable. Only the National Cancer Institute of the United States has made recommendations for the standardization of EBV DNA testing 1, 37. Moreover, ELISA and chemiluminescence methods are more suitable for large-scale screening of NPC. Therefore, we used a combined antibody detection method to establish a risk prediction model for NPC in this study.

However, this study has some limitations that should be acknowledged. First, since the samples in this study were exclusively obtained from a renowned large comprehensive tertiary hospital in Zhejiang Province, early-stage patients may have already received treatment at local medical institutions. As a result, the cases treated at this hospital predominantly consisted of intermediate and advanced-stage patients, potentially introducing a certain degree of case selection bias. To minimize the impact of selection bias, future research will involve multi-center collaboration for case collection and statistical analysis, aiming to enhance the representativeness of the study and the reliability of the results. Second, this research has limitations regarding the sample size of clinical data: although statistical analysis was performed on the collected NPC samples, the relatively small sample size may restrict the generalizability and representativeness of the findings. Future research could expand the sample size and include more diverse clinical subtypes and stages of NPC to further validate the universality of the risk prediction model across varied patient populations and to provide stronger evidence supporting its clinical application value.

## Conclusion

The combination of P85-Ab with VCA-IgA and Rta-IgG showed excellent performance in terms of sensitivity and specificity, which highlighted its potential as a large-scale NPC screening method in regions with a relatively low incidence of NPC. However, large-scale prospective studies are warranted to validate the predictive performance of this combination.

## Figures and Tables

**Table 1:** Clinical characteristics of individuals in the NPC and control cohorts.

Characteristics	Entire study (479)	Clinical Stage			NPC (81)	Control (398)	χ^2^ or U Value	p Valuec
		I or II (6)	III or IV (65)	p Valued				
Age (years), median (range)	49 (27-88)	56 (37-70)	57 (27-81)	0.852a	57 (27-81)	47 (27-88)	-3.701a	<0.001
Gender, n (%)							3.497b	0.061
Female	115 (24.0%)	3 (50.0%)	17 (26.2%)	0.442b	26 (32.1%)	89 (22.4%)		
Male	364 (76.0%)	3 (50.0%)	48 (73.8%)		55 (67.9%)	309 (77.6%)		
EA-IgA, median (range)	0.15 (0.01-5.11)	0.78 (0.03-1.64)	0.69 (0.07-5.11)	0.710a	0.61 (0.03-5.11)	0.12 (0.01-2.60)	-9.431a	<0.001
Rta-IgG, median (range)	0.21 (0.03-4.12)	0.80 (0.19-2.84)	1.21 (0.05-4.12)	0.918a	0.95 (0.05-4.12)	0.19 (0.03-3.22)	-8.668a	<0.001
P85-Ab, median (range)	0.01 (0.00-639.48)	81.84 (5.14-292.57)	44.47 (0.01-639.48)	0.591a	43.32 (0.01-639.48)	0.01 (0.00-13.01)	-13.898a	<0.001
VCA-IgG, n (%)							0.669b	0.413
Positive	463 (96.7%)	6 (100%)	64 (98.5%)	1.0b	80 (98.8%)	383 (96.2%)		
Negative	16 (3.3%)	0 (0%)	1 (1.5%)		1 (1.2%)	15 (3.8%)		
VCA-IgM, n (%)							0.953b	0.329
Positive	13 (2.7%)	0 (0%)	4 (6.2%)	1.0b	4 (4.9%)	9 (2.3%)		
Negative	466 (97.3%)	6 (100%)	61 (93.8%)		77 (95.1%)	389 (97.7%)		
VCA-IgA, n (%)							262.25b	<0.001
Positive	103 (21.5%)	4 (66.7%)	60 (92.3%)	0.104b	72 (88.9%)	31 (7.8%)		
Negative	376 (78.5%)	2 (33.3%)	5 (7.7%)		9 (11.1%)	367 (92.2%)		

^a^ Mann-Whitney U test; ^b^ χ^2^ test; ^c^ *P* value comes from NPC group and control group. ^d^ *P* value comes from early stage (I or II) and advanced stage (III or IV) groups.

**Table 2:** Receiver operating characteristic curve data of P85-Ab, VCA-IgA, Rta-IgG and EA-IgA, for prediction of nasopharyngeal carcinoma.

						VS. Combined	
Variables	AUC (95% CI)	Cut-off Value	*P*-Value	Sensitivity (%)	Specificity (%)	Z-Value	*P*-Value
Combined	0.977 (0.959-0.988)	>0.148	<0.0001	91.36	99.25	-	-
P85-Ab	0.949 (0.925-0.967)	>0.24	<0.0001	86.42	99.50	1.906	0.088
VCA-IgA	0.905 (0.876-0.930)	>1.0	<0.0001	88.89	92.21	4.426	<0.0001
Rta-IgG	0.804 (0.766-0.839)	>0.7	<0.0001	56.79	95.98	5.735	<0.0001
EA-IgA	0.832 (0.796-0.865)	>0.39	<0.0001	61.73	90.20	5.321	<0.0001
